# Crystal structure of strontium and barium acesulafame (6-methyl-4-oxo-4*H*-1,2,3-oxa­thia­zin-3-ide 2,2-dioxide)

**DOI:** 10.1107/S2056989018006059

**Published:** 2018-04-24

**Authors:** Alexander Y. Nazarenko

**Affiliations:** a Chemistry Department, SUNY Buffalo State, 1300 Elmwood Ave, Buffalo, NY 14222, USA

**Keywords:** crystal structure, acesulafame, 6-methyl-4-oxo-4*H*-1,2,3-oxa­thia­zin-3-ide 2,2-dioxide, strontium, barium

## Abstract

Both strontium and barium acesulfames, namely poly[aqua­bis­(μ_3_-6-methyl-2,2-dioxo-1,2λ^6^,3-oxa­thia­zin-4-olato)strontium(II)], [Sr(C_4_H_4_NO_4_S)_2_(H_2_O)]_*n*_, and the barium(II) analogue, [Ba(C_4_H_4_NO_4_S)_2_(H_2_O)]_*n*_, crystallize in nearly identical isotypic forms, with barium–oxygen inter­atomic distances being longer due to the larger ionic radius of the barium(II) ion. The coordination number of the metal ion is 9; the coordination polyhedra can be described as distorted capped square anti­prisms [Johnson solid **J10**; Johnson (1966). *Can. J. Math.* **18**, 169–200]. The conformation of the acesulafame ions is a distorted envelope with an out-of-plane S atom. Metal and acesulfame ions are assembled into infinitive chains along the [100] axis. These chains are connected *via* hydrogen bonds into a three-dimensional network.

## Abstract 2

Strontium and barium acesulfames crystallize in nearly identical isotypic forms, with barium–oxygen inter­atomic distances being longer due to the larger ionic radius of the barium(II) ion. The conformation of the acesulafame ions is a distorted envelope with an out-of-plane S atom. Metal and acesulfame ions are assembled in infinitive chains along the [100] axis. These chains are connected *via* hydrogen bonds into a three-dimensional network.

## Chemical context   

Acesulfame is one of the most common sweeteners; usually it is used in the form of a potassium salt. Salts with all alkali metals, ammonium, magnesium, and calcium ions, as well as its protonated mol­ecular form, are also known. The almost identical crystal structures of the strontium and barium salts are reported here.
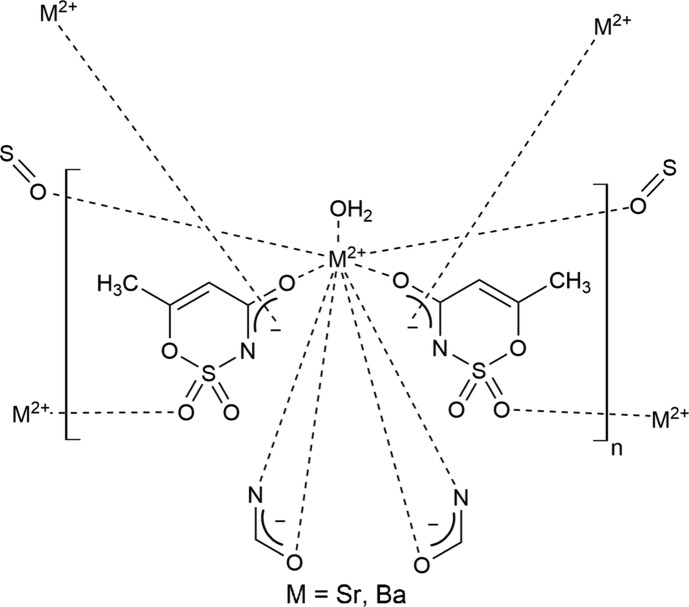


## Structural commentary   

**Figure 1:**
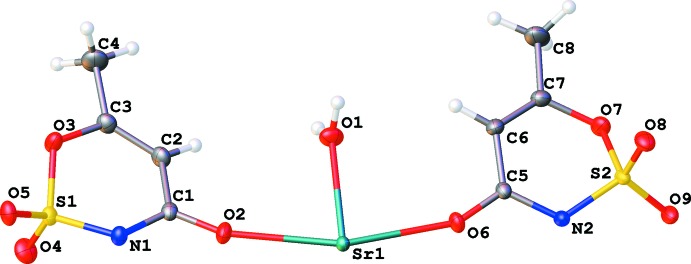
The numbering scheme of strontium acesulfame monohydrate, shown with 50% probability displacement elipsoids.

**Figure 2:**
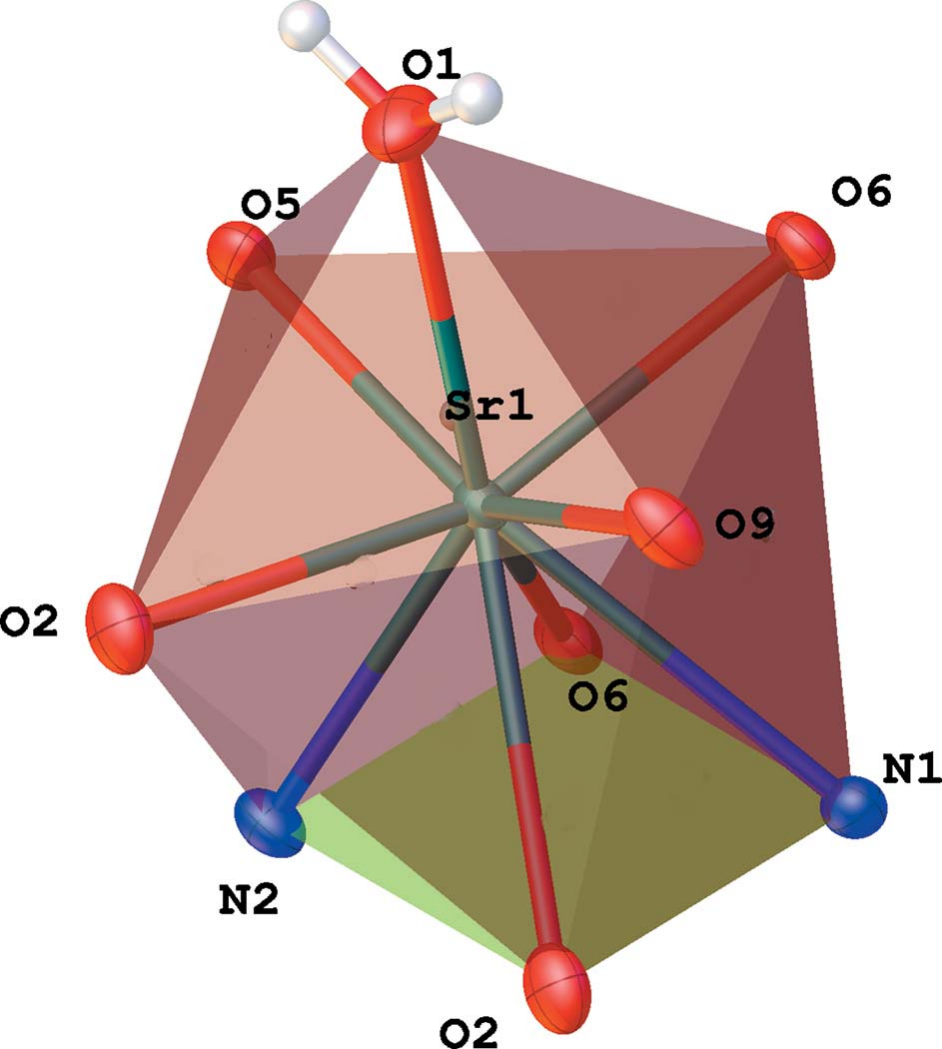
The coordination polyhedron of the Sr metal ion. Symmetry codes for the base rectangle atoms are: N1 and O2 at (−*x* + 1, −*y* + 1, −*z* + 1), N2 and O6 at (−*x* + 2, −*y* + 1, −*z* + 1). For the upper rectangle: O9 is at (*x* − 1, *y*, *z*) and O5 is at (*x* + 1, *y*, *z*).

Both the strontium and barium compounds crystallize in nearly identical isotypic forms (Fig. 1 ▸), with the barium–oxygen inter­atomic distances being longer due to the larger ionic radius of the barium(II) ion (Tables 1 ▸ and 2 ▸). Because of the similarity of the structures, representations would be visually identical, and all figures are shown for the strontium salt only. In both cases, the coordination sphere of the metal ion contains a water mol­ecule, two O atoms from carbonyl groups, two O atoms from sulfonyl fragments, and two bidentate amide groups coordinated through both their carbonyl group and the adjacent deprotonated N atom (Fig. 2 ▸). The total coordination number is nine. This coordination polyhedron can be described as a distorted capped square anti­prism. The corresponding ideal polyhedron is a gyro­elongated square pyramid, a Johnson solid **J10** (Johnson, 1966 ▸). In this case, the base of the polyhedron is not a square but a rectangle formed by two N and two O atoms from two bidentate amide groups. Nitro­gen–oxygen distances within each of the groups are practically the same: N1^i^⋯O2^i^ = 2.232 (2) Å and N2^iii^⋯O6^iii^ = 2.233 (2) Å (Sr); and N1^i^⋯O2^i^ = 2.233 (3) Å and N2^iii^⋯O6^iii^ = 2.239 (2) Å (Ba). The inter­atomic separations between these groups are much longer: N1^i^⋯O6^iii^ = 3.0609 (19) (Sr) and 3.174 (3) Å (Ba), and N2^iii^⋯O2^i^ = 3.0787 (18) (Sr) and 3.184 (3) Å (Ba) [symmetry codes: (i) −*x* + 1, −*y* + 1, −*z* + 1; (iii) −*x* + 2, −*y* + 1, −*z* + 1.]

Deviations from exact right angles are around 5° for the Sr and 1.5° for the Ba structure. The upper square of the coordination polyhedron is less distorted, with angles very close (mostly within 1°) to 90°. The two average planes of the base rectangle and upper square are nearly coplanar, with the angles between them being 1.17 (4) (Sr) and 0.99 (6)° (Ba). The line between the metal ions and the capping oxygen of the water mol­ecules are nearly perpendicular to these planes: the angles between the upper plane normal and the connecting line are 4.90 (4) (Sr) and 5.58 (6)° (Ba).

**Figure 3:**
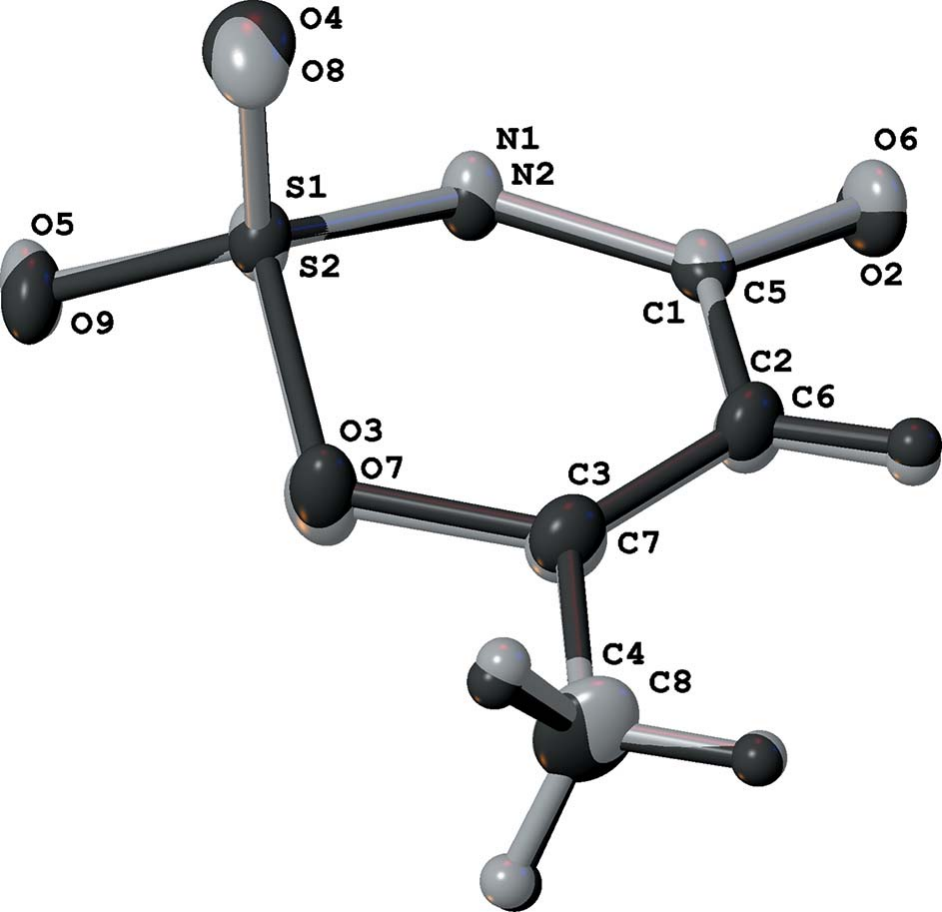
Overlay of the two acesulafame ions in the Sr structure.

**Figure 4:**
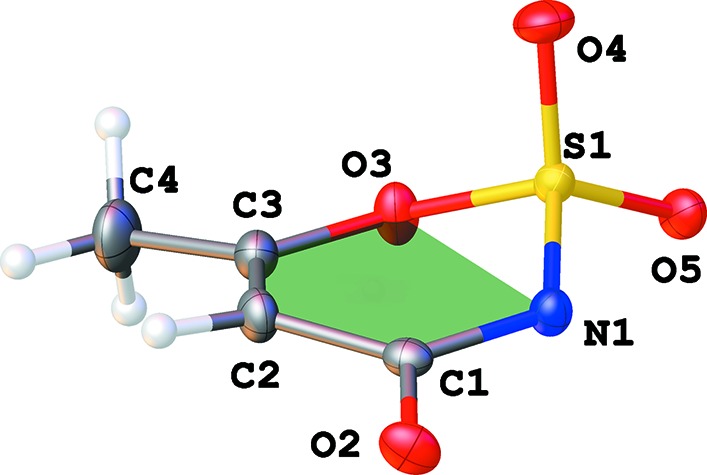
View of the acesulafame anion in the Sr structure. Deviations from the average plane of five atoms in six-membered ring are 0.031 (N1), −0.010 (O3), −0.011 (C3), +0.042 (C2), −0.052 (C1) and 0.5377 (15) Å (S1).

The geometries of the two acesulfame anions in each structure are very similar to each other (Fig. 3 ▸), as well as to those of previously reported compounds. The six-membered rings have only one atom with a tetra­hedral environment (S). The other five atoms deviate only slightly from their average planes (Fig. 4 ▸). This conformation can be described as an envelope, slightly distorted toward a boat.

## Supra­molecular features   

**Figure 5:**
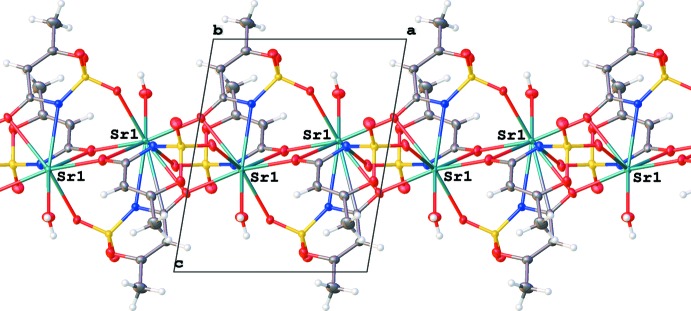
The infinite chain of metal ions and acesulafame anions along the [100] axis. The view is along the [010] vector.

**Figure 6:**
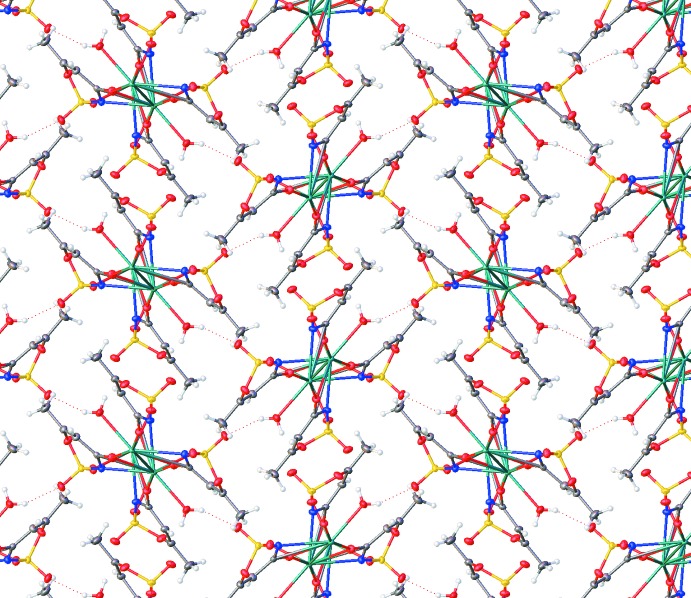
The packing of Sr acesulafame, viewed along the [100] axis.

Each acesulfame anion is connected to three strontium (or barium) ions *via* a bridging O atom of the amide carbonyl group, an N atom of this group, and one of the O atoms of a sulfonyl group. Each metal ion is directly connected to six acesulfame anions. As a result, metal ions and acesulfame anions form infinite chains along the [100] axis (Fig. 5 ▸). The O atoms of the two sulfonyl groups that are not connected to metal ions form, instead, strong hydrogen bonds with both H atoms of the water mol­ecule. These hydrogen bonds (Tables 3 ▸ and 4 ▸) connect each chain to four neighboring parellel chains, thus creating a three-dimensional assembly (Fig. 6 ▸). There are also two short C—H⋯O contacts (Tables 3 ▸ and 4 ▸) which may additionally stabilize the crystal structures.

## Database survey   

There are over 40 acesulfame structures deposited in the Cambridge Structural Database (CSD; Groom *et al.*, 2016 ▸; Version 5.38). Of these structures, alkali metal, ammonium, and thallium salts FEQPUP (Piro *et al.*, 2017 ▸), KMTOZD (Paulus, 1975 ▸), SUQTOP, SURCIT and SURCOZ (Piro *et al.*, 2015 ▸), TOFPEL (Echeverría *et al.*, 2014 ▸), OCAHUY (Baran *et al.*, 2015 ▸), magnesium salt XAGVAF (Piro *et al.*, 2016 ▸), calcium salt EXUCOR (Demirtas *et al.*, 2012 ▸), and protonated forms WURMOM and WURMOM01 (Velaga *et al.*, 2010 ▸) are closely related to the structures of the title compounds. Several other structures describe coordination compounds with transition-metal ions and various salts of bulky organic cations.

## Synthesis and crystallization   

The protonated form of acesulfame, 6-methyl-1,2,3-oxa­thia­zin-4(3*H*)-one 2,2-dioxide, was synthesized following a published procedure (Velaga *et al.*, 2010 ▸). The starting compound, potassium acesulfame, was obtained at a stated purity of 99% and no attempt was made at further purification. 2 g (0.01 mol) of it were dissolved in water (10 ml), acidified with 10 ml of 6 *M* HCl, and extracted with several portions of methyl­ene chloride (15 ml each). Evaporation of the methyl­ene chloride extract resulted in crystals of protonated acesulfame (identified by X-ray diffraction; CSD refcode WURMOM). Both strontium oxide (100 mg, 0.001 mol) and barium oxide (150 mg, 0.001 mol) were treated with a small amount of water (*ca* 10 ml), forming the corresponding hydroxides. Stoichiometric amounts (0.002 mol, 320 mg) of 6-methyl-1,2,3-oxa­thia­zin-4(3*H*)-one 2,2-dioxide were added under gentle heating to neutralize the alkaline solutions. Slow evaporation of the filtrated solutions resulted in colorless crystals (around 100 mg), some of which were suitable for X-ray investigation. No attempts to optimize the reaction conditions or to recover more material were made. Several crystals were tested; the best results obtained are reported here. FT-IR-ATR, Sr–acesulfame (cm^−1^): 3620, 3550 (H_2_O), 1641 (amide), 1555, 1173 (SO_2_), 938.

## Refinement   

Crystal data, data collection and structure refinement details are summarized in Table 5 ▸. All H atoms of water mol­ecules are refined in isotropic approximation. All other H atoms are refined with riding coordinates; methyl H atoms are refined as rotating idealized methyl groups and with *U*_iso_(H) = 1.5*U*_iso_(C).

## Supplementary Material

Crystal structure: contains datablock(s) Ba, Sr. DOI: 10.1107/S2056989018006059/zl2727sup1.cif

Structure factors: contains datablock(s) Sr. DOI: 10.1107/S2056989018006059/zl2727Srsup2.hkl

Supporting information file. DOI: 10.1107/S2056989018006059/zl2727Srsup4.cdx

Structure factors: contains datablock(s) Ba. DOI: 10.1107/S2056989018006059/zl2727Basup3.hkl

CCDC references: 1838460, 1838459

Additional supporting information:  crystallographic information; 3D view; checkCIF report

## Figures and Tables

**Table 1:** Selected bond lengths (Å) for Sr[Chem scheme1]

Sr1—O1	2.6000 (14)	Sr1—O6^iii^	2.8070 (13)
Sr1—O2^i^	2.9039 (14)	Sr1—O9^iv^	2.5782 (12)
Sr1—O2	2.5026 (12)	Sr1—N1^i^	2.7553 (15)
Sr1—O5^ii^	2.5790 (13)	Sr1—N2^iii^	2.7447 (14)
Sr1—O6	2.5116 (12)		

Symmetry codes: (i) 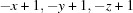; (ii) 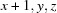; (iii) 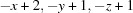; (iv) 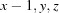.

**Table 2:** Selected bond lengths (Å) for Ba[Chem scheme1]

Ba1—O1	2.732 (2)	Ba1—O6^iii^	2.9192 (18)
Ba1—O2^i^	2.9714 (19)	Ba1—O9^iv^	2.7478 (18)
Ba1—O2	2.6812 (17)	Ba1—N1^i^	2.921 (2)
Ba1—O5^ii^	2.7560 (18)	Ba1—N2^iii^	2.9077 (19)
Ba1—O6	2.6788 (16)		

Symmetry codes: (i) 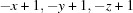; (ii) 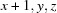; (iii) 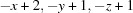; (iv) 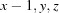.

**Table 3:** Hydrogen-bond geometry (Å, °) for Sr[Chem scheme1]

*D*—H⋯*A*	*D*—H	H⋯*A*	*D*⋯*A*	*D*—H⋯*A*
O1—H1*A*⋯O4^v^	0.79 (3)	2.18 (3)	2.899 (2)	151 (3)
O1—H1*B*⋯O8^vi^	0.83 (3)	2.22 (3)	2.9850 (19)	152 (3)
C4—H4*B*⋯O5^v^	0.98	2.37	3.273 (3)	153
C6—H6⋯O8^vi^	0.95	2.47	3.318 (2)	148

Symmetry codes: (v) 
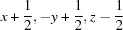
; (vi) 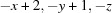.

**Table 4:** Hydrogen-bond geometry (Å, °) for Ba[Chem scheme1]

*D*—H⋯*A*	*D*—H	H⋯*A*	*D*⋯*A*	*D*—H⋯*A*
O1—H1*A*⋯O4^v^	0.74 (4)	2.12 (4)	2.829 (3)	162 (4)
O1—H1*B*⋯O8^vi^	0.74 (4)	2.20 (4)	2.889 (3)	156 (4)
C4—H4*B*⋯O5^v^	0.98	2.44	3.361 (4)	155
C6—H6⋯O8^vi^	0.95	2.52	3.357 (3)	147

Symmetry codes: (v) 
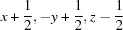
; (vi) 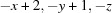.

**Table 5:** Experimental details

	Sr complex	Ba complex
Crystal data		
Chemical formula	[Sr(C_4_H_4_NO_4_S)_2_(H_2_O)]	[Ba(C_4_H_4_NO_4_S)_2_(H_2_O)]
*M*_r_	429.92	479.64
Crystal system, space group	Monoclinic, *P*2_1_/*n*	Monoclinic, *P*2_1_/*n*
Temperature (K)	173	173
*a*, *b*, *c* (Å)	7.9695 (3), 18.5217 (8), 9.7310 (4)	8.2086 (5), 18.8899 (11), 9.8999 (6)
β (°)	99.651 (1)	99.5197 (10)
*V* (Å^3^)	1416.05 (10)	1513.93 (16)
*Z*	4	4
Radiation type	Mo *K*α	Mo *K*α
μ (mm^−1^)	4.15	2.94
Crystal size (mm)	0.55 × 0.13 × 0.07	0.52 × 0.21 × 0.20

Data collection		
Diffractometer	Bruker PHOTON-100 CMOS	Bruker PHOTON-100 CMOS
Absorption correction	Numerical (*SADABS*; Krause *et al.*, 2015 ▸)	Numerical (*SADABS*; Krause *et al.*, 2015 ▸)
*T*_min_, *T*_max_	0.228, 0.797	0.309, 0.635
No. of measured, independent and observed [*I* > 2σ(*I*)] reflections	54583, 5414, 4548	41654, 6324, 5218
*R*_int_	0.043	0.048
(sin θ/λ)_max_ (Å^−1^)	0.771	0.794

Refinement		
*R*[*F* ^2^ > 2σ(*F* ^2^)], *wR*(*F* ^2^), *S*	0.027, 0.062, 1.04	0.032, 0.059, 1.09
No. of reflections	5414	6324
No. of parameters	209	209
H-atom treatment	H atoms treated by a mixture of independent and constrained refinement	H atoms treated by a mixture of independent and constrained refinement
Δρ_max_, Δρ_min_ (e Å^−3^)	0.73, −0.46	1.02, −0.61

Computer programs: *APEX2* and *SAINT* (Bruker, 2013 ▸), *SHELXT* (Sheldrick, 2015*a* ▸), *SHELXL2016* (Sheldrick, 2015*b* ▸) and *OLEX2* (Dolomanov *et al.*, 2009 ▸).

## supplementary crystallographic information

### Poly[aquabis(µ3-6-methyl-2,2-dioxo-1,2λ6,3-oxathiazin-4-olato)strontium(II)] (Sr) Crystal data

**Table:**

[Sr(C_4_H_4_NO_4_S)_2_(H_2_O)]	*F*(000) = 856
*M**_r_* = 429.92	*D*_x_ = 2.017 Mg m^−^^3^
Monoclinic, *P*2_1_/*n*	Mo *K*α radiation, λ = 0.71073 Å
*a* = 7.9695 (3) Å	Cell parameters from 9082 reflections
*b* = 18.5217 (8) Å	θ = 3.1–33.3°
*c* = 9.7310 (4) Å	µ = 4.15 mm^−^^1^
β = 99.651 (1)°	*T* = 173 K
*V* = 1416.05 (10) Å^3^	Needle, colourless
*Z* = 4	0.55 × 0.13 × 0.07 mm

### Poly[aquabis(µ3-6-methyl-2,2-dioxo-1,2λ6,3-oxathiazin-4-olato)strontium(II)] (Sr) Data collection

**Table:**

Bruker PHOTON-100 CMOS diffractometer	4548 reflections with *I* > 2σ(*I*)
φ and ω scans	*R*_int_ = 0.043
Absorption correction: numerical (SADABS; Krause *et al.*, 2015)	θ_max_ = 33.2°, θ_min_ = 3.1°
*T*_min_ = 0.228, *T*_max_ = 0.797	*h* = −12→12
54583 measured reflections	*k* = −28→28
5414 independent reflections	*l* = −14→14

### Poly[aquabis(µ3-6-methyl-2,2-dioxo-1,2λ6,3-oxathiazin-4-olato)strontium(II)] (Sr) Refinement

**Table:**

Refinement on *F*^2^	Primary atom site location: structure-invariant direct methods
Least-squares matrix: full	Secondary atom site location: difference Fourier map
*R*[*F*^2^ > 2σ(*F*^2^)] = 0.027	Hydrogen site location: mixed
*wR*(*F*^2^) = 0.062	H atoms treated by a mixture of independent and constrained refinement
*S* = 1.04	*w* = 1/[σ^2^(*F*_o_^2^) + (0.0247*P*)^2^ + 1.3759*P*] where *P* = (*F*_o_^2^ + 2*F*_c_^2^)/3
5414 reflections	(Δ/σ)_max_ = 0.001
209 parameters	Δρ_max_ = 0.73 e Å^−^^3^
0 restraints	Δρ_min_ = −0.46 e Å^−^^3^

### Poly[aquabis(µ3-6-methyl-2,2-dioxo-1,2λ6,3-oxathiazin-4-olato)strontium(II)] (Sr) Special details

**Table:**

Geometry. All esds (except the esd in the dihedral angle between two l.s. planes) are estimated using the full covariance matrix. The cell esds are taken into account individually in the estimation of esds in distances, angles and torsion angles; correlations between esds in cell parameters are only used when they are defined by crystal symmetry. An approximate (isotropic) treatment of cell esds is used for estimating esds involving l.s. planes.

### Poly[aquabis(µ3-6-methyl-2,2-dioxo-1,2λ6,3-oxathiazin-4-olato)strontium(II)] (Sr) Fractional atomic coordinates and isotropic or equivalent isotropic displacement parameters (Å^2^)

**Table:**

	*x*	*y*	*z*	*U*_iso_*/*U*_eq_	
Sr1	0.73894 (2)	0.46960 (2)	0.43922 (2)	0.01161 (4)	
S1	0.07248 (5)	0.32528 (2)	0.53917 (4)	0.01432 (7)	
S2	1.37055 (5)	0.52355 (2)	0.16245 (4)	0.01457 (8)	
O1	0.69696 (19)	0.37734 (8)	0.23495 (15)	0.0236 (3)	
H1A	0.703 (4)	0.3346 (16)	0.232 (3)	0.038 (8)*	
H1B	0.656 (4)	0.3914 (17)	0.155 (3)	0.049 (9)*	
O2	0.46642 (16)	0.41317 (7)	0.48048 (14)	0.0216 (3)	
O3	0.04339 (16)	0.28794 (7)	0.38723 (14)	0.0207 (3)	
O4	0.11859 (18)	0.26877 (7)	0.63821 (14)	0.0247 (3)	
O5	−0.08823 (16)	0.35905 (7)	0.54351 (14)	0.0200 (2)	
O6	1.00611 (16)	0.48946 (7)	0.33932 (13)	0.0185 (2)	
O7	1.34454 (16)	0.44775 (8)	0.07948 (14)	0.0218 (3)	
O8	1.31994 (17)	0.57900 (8)	0.06234 (13)	0.0223 (3)	
O9	1.54716 (15)	0.52276 (7)	0.22320 (13)	0.0197 (2)	
N1	0.21644 (18)	0.38308 (8)	0.53881 (15)	0.0159 (3)	
N2	1.25495 (18)	0.51967 (8)	0.27867 (14)	0.0162 (3)	
C1	0.3450 (2)	0.36951 (9)	0.46730 (17)	0.0149 (3)	
C2	0.3318 (2)	0.30990 (10)	0.36758 (18)	0.0191 (3)	
H2	0.4300	0.2956	0.3311	0.023*	
C3	0.1853 (2)	0.27495 (10)	0.32641 (18)	0.0188 (3)	
C4	0.1419 (3)	0.22384 (12)	0.2083 (2)	0.0310 (4)	
H4A	0.0983	0.1789	0.2419	0.046*	
H4B	0.2441	0.2135	0.1680	0.046*	
H4C	0.0548	0.2454	0.1370	0.046*	
C5	1.1042 (2)	0.48462 (9)	0.25133 (17)	0.0142 (3)	
C6	1.0638 (2)	0.43825 (10)	0.12897 (17)	0.0175 (3)	
H6	0.9511	0.4203	0.1040	0.021*	
C7	1.1800 (2)	0.42032 (10)	0.05129 (17)	0.0186 (3)	
C8	1.1604 (3)	0.36965 (12)	−0.0686 (2)	0.0312 (4)	
H8A	1.1812	0.3954	−0.1521	0.047*	
H8B	1.0446	0.3500	−0.0848	0.047*	
H8C	1.2424	0.3301	−0.0480	0.047*	

### Poly[aquabis(µ3-6-methyl-2,2-dioxo-1,2λ6,3-oxathiazin-4-olato)strontium(II)] (Sr) Atomic displacement parameters (Å^2^)

**Table:**

	*U*^11^	*U*^22^	*U*^33^	*U*^12^	*U*^13^	*U*^23^
Sr1	0.01046 (6)	0.01363 (7)	0.01116 (6)	−0.00146 (5)	0.00300 (4)	−0.00080 (5)
S1	0.01438 (17)	0.01221 (16)	0.01734 (18)	−0.00014 (13)	0.00547 (13)	−0.00002 (13)
S2	0.01118 (16)	0.0224 (2)	0.01036 (16)	−0.00041 (14)	0.00245 (12)	−0.00008 (14)
O1	0.0324 (7)	0.0183 (6)	0.0196 (6)	−0.0036 (5)	0.0034 (5)	−0.0044 (5)
O2	0.0156 (6)	0.0261 (7)	0.0230 (6)	−0.0074 (5)	0.0032 (5)	0.0018 (5)
O3	0.0161 (6)	0.0236 (6)	0.0235 (6)	−0.0044 (5)	0.0061 (5)	−0.0086 (5)
O4	0.0291 (7)	0.0179 (6)	0.0278 (7)	0.0027 (5)	0.0068 (5)	0.0079 (5)
O5	0.0156 (5)	0.0187 (6)	0.0280 (7)	0.0015 (5)	0.0101 (5)	0.0016 (5)
O6	0.0160 (5)	0.0223 (6)	0.0189 (6)	−0.0016 (5)	0.0085 (5)	−0.0008 (5)
O7	0.0185 (6)	0.0283 (7)	0.0202 (6)	−0.0013 (5)	0.0077 (5)	−0.0082 (5)
O8	0.0196 (6)	0.0301 (7)	0.0168 (6)	0.0013 (5)	0.0020 (5)	0.0068 (5)
O9	0.0113 (5)	0.0322 (7)	0.0153 (5)	−0.0004 (5)	0.0016 (4)	0.0019 (5)
N1	0.0139 (6)	0.0156 (6)	0.0190 (6)	−0.0005 (5)	0.0049 (5)	−0.0018 (5)
N2	0.0132 (6)	0.0233 (7)	0.0124 (6)	−0.0028 (5)	0.0029 (5)	−0.0021 (5)
C1	0.0129 (6)	0.0160 (7)	0.0155 (7)	0.0003 (5)	0.0014 (5)	0.0042 (5)
C2	0.0178 (7)	0.0211 (8)	0.0201 (8)	0.0020 (6)	0.0077 (6)	−0.0022 (6)
C3	0.0213 (8)	0.0174 (8)	0.0190 (8)	0.0012 (6)	0.0066 (6)	−0.0017 (6)
C4	0.0377 (11)	0.0292 (10)	0.0274 (10)	−0.0034 (9)	0.0092 (8)	−0.0135 (8)
C5	0.0120 (6)	0.0177 (7)	0.0130 (7)	0.0006 (5)	0.0024 (5)	0.0028 (5)
C6	0.0162 (7)	0.0220 (8)	0.0138 (7)	−0.0033 (6)	0.0008 (6)	−0.0008 (6)
C7	0.0217 (8)	0.0199 (8)	0.0137 (7)	−0.0022 (6)	0.0016 (6)	−0.0014 (6)
C8	0.0420 (12)	0.0308 (10)	0.0223 (9)	−0.0102 (9)	0.0099 (8)	−0.0113 (8)

### Poly[aquabis(µ3-6-methyl-2,2-dioxo-1,2λ6,3-oxathiazin-4-olato)strontium(II)] (Sr) Geometric parameters (Å, º)

**Table:**

Sr1—Sr1^i^	4.2816 (3)	O5—Sr1^iv^	2.5789 (13)
Sr1—O1	2.6000 (14)	O6—Sr1^i^	2.8070 (13)
Sr1—O2^ii^	2.9039 (14)	O6—C5	1.2556 (19)
Sr1—O2	2.5026 (12)	O7—C7	1.390 (2)
Sr1—O5^iii^	2.5790 (13)	O9—Sr1^iii^	2.5782 (12)
Sr1—O6	2.5116 (12)	N1—Sr1^ii^	2.7553 (15)
Sr1—O6^i^	2.8070 (13)	N1—C1	1.355 (2)
Sr1—O9^iv^	2.5782 (12)	N2—Sr1^i^	2.7448 (14)
Sr1—N1^ii^	2.7553 (15)	N2—C5	1.352 (2)
Sr1—N2^i^	2.7447 (14)	C1—Sr1^ii^	3.2186 (17)
Sr1—C1^ii^	3.2186 (17)	C1—C2	1.462 (2)
Sr1—C5^i^	3.1761 (16)	C2—H2	0.9500
S1—O3	1.6134 (13)	C2—C3	1.336 (2)
S1—O4	1.4285 (13)	C3—C4	1.484 (3)
S1—O5	1.4323 (13)	C4—H4A	0.9800
S1—N1	1.5697 (15)	C4—H4B	0.9800
S2—O7	1.6156 (14)	C4—H4C	0.9800
S2—O8	1.4264 (13)	C5—Sr1^i^	3.1762 (16)
S2—O9	1.4324 (13)	C5—C6	1.460 (2)
S2—N2	1.5755 (14)	C6—H6	0.9500
O1—H1A	0.79 (3)	C6—C7	1.332 (2)
O1—H1B	0.84 (3)	C7—C8	1.485 (3)
O2—Sr1^ii^	2.9040 (14)	C8—H8A	0.9800
O2—C1	1.251 (2)	C8—H8B	0.9800
O3—C3	1.383 (2)	C8—H8C	0.9800
			
O1—Sr1—Sr1^i^	112.25 (3)	O4—S1—O5	115.48 (8)
O1—Sr1—O2^ii^	133.71 (4)	O4—S1—N1	113.32 (8)
O1—Sr1—O6^i^	138.44 (4)	O5—S1—O3	102.85 (7)
O1—Sr1—N1^ii^	135.12 (5)	O5—S1—N1	111.08 (8)
O1—Sr1—N2^i^	142.25 (5)	N1—S1—O3	106.45 (7)
O1—Sr1—C1^ii^	144.92 (5)	O8—S2—O7	106.65 (8)
O1—Sr1—C5^i^	151.65 (5)	O8—S2—O9	115.79 (8)
O2—Sr1—Sr1^i^	153.49 (3)	O8—S2—N2	113.00 (8)
O2^ii^—Sr1—Sr1^i^	107.12 (3)	O9—S2—O7	103.43 (8)
O2—Sr1—O1	81.04 (5)	O9—S2—N2	110.83 (8)
O2—Sr1—O2^ii^	74.08 (5)	N2—S2—O7	106.12 (7)
O2—Sr1—O5^iii^	91.48 (4)	Sr1—O1—H1A	133 (2)
O2—Sr1—O6	159.74 (4)	Sr1—O1—H1B	119 (2)
O2—Sr1—O6^i^	121.71 (4)	H1A—O1—H1B	107 (3)
O2—Sr1—O9^iv^	83.02 (4)	Sr1—O2—Sr1^ii^	105.92 (5)
O2—Sr1—N1^ii^	120.35 (4)	C1—O2—Sr1	158.55 (12)
O2—Sr1—N2^i^	74.94 (4)	C1—O2—Sr1^ii^	92.84 (10)
O2^ii^—Sr1—C1^ii^	22.85 (4)	C3—O3—S1	117.69 (11)
O2—Sr1—C1^ii^	96.57 (4)	S1—O5—Sr1^iv^	139.14 (7)
O2^ii^—Sr1—C5^i^	72.26 (4)	Sr1—O6—Sr1^i^	107.09 (4)
O2—Sr1—C5^i^	100.00 (4)	C5—O6—Sr1^i^	95.14 (10)
O5^iii^—Sr1—Sr1^i^	70.73 (3)	C5—O6—Sr1	156.71 (12)
O5^iii^—Sr1—O1	76.75 (5)	C7—O7—S2	117.00 (11)
O5^iii^—Sr1—O2^ii^	140.87 (4)	S2—O9—Sr1^iii^	138.38 (8)
O5^iii^—Sr1—O6^i^	69.04 (4)	S1—N1—Sr1^ii^	139.48 (8)
O5^iii^—Sr1—N1^ii^	134.61 (4)	C1—N1—Sr1^ii^	97.17 (10)
O5^iii^—Sr1—N2^i^	75.24 (4)	C1—N1—S1	119.36 (12)
O5^iii^—Sr1—C1^ii^	138.28 (4)	S2—N2—Sr1^i^	143.15 (8)
O5^iii^—Sr1—C5^i^	74.90 (4)	C5—N2—Sr1^i^	95.61 (9)
O6^i^—Sr1—Sr1^i^	34.10 (2)	C5—N2—S2	119.20 (12)
O6—Sr1—Sr1^i^	38.80 (3)	O2—C1—Sr1^ii^	64.31 (9)
O6—Sr1—O1	79.00 (4)	O2—C1—N1	117.77 (16)
O6^i^—Sr1—O2^ii^	87.68 (4)	O2—C1—C2	121.63 (15)
O6—Sr1—O2^ii^	123.18 (4)	N1—C1—Sr1^ii^	58.14 (9)
O6—Sr1—O5^iii^	80.62 (4)	N1—C1—C2	120.30 (15)
O6—Sr1—O6^i^	72.91 (4)	C2—C1—Sr1^ii^	153.07 (11)
O6—Sr1—O9^iv^	93.08 (4)	C1—C2—H2	119.1
O6—Sr1—N1^ii^	77.19 (4)	C3—C2—C1	121.80 (15)
O6—Sr1—N2^i^	120.16 (4)	C3—C2—H2	119.1
O6^i^—Sr1—C1^ii^	71.99 (4)	O3—C3—C4	109.98 (16)
O6—Sr1—C1^ii^	101.74 (4)	C2—C3—O3	121.56 (16)
O6—Sr1—C5^i^	95.93 (4)	C2—C3—C4	128.27 (17)
O6^i^—Sr1—C5^i^	23.19 (4)	C3—C4—H4A	109.5
O9^iv^—Sr1—Sr1^i^	122.73 (3)	C3—C4—H4B	109.5
O9^iv^—Sr1—O1	69.20 (5)	C3—C4—H4C	109.5
O9^iv^—Sr1—O2^ii^	69.51 (4)	H4A—C4—H4B	109.5
O9^iv^—Sr1—O5^iii^	145.95 (4)	H4A—C4—H4C	109.5
O9^iv^—Sr1—O6^i^	140.95 (4)	H4B—C4—H4C	109.5
O9^iv^—Sr1—N1^ii^	74.71 (4)	O6—C5—Sr1^i^	61.67 (9)
O9^iv^—Sr1—N2^i^	134.15 (4)	O6—C5—N2	117.76 (15)
O9^iv^—Sr1—C1^ii^	75.76 (4)	O6—C5—C6	121.39 (15)
O9^iv^—Sr1—C5^i^	139.15 (4)	N2—C5—Sr1^i^	59.32 (8)
N1^ii^—Sr1—Sr1^i^	67.12 (3)	N2—C5—C6	120.67 (14)
N1^ii^—Sr1—O2^ii^	46.36 (4)	C6—C5—Sr1^i^	158.19 (11)
N1^ii^—Sr1—O6^i^	66.77 (4)	C5—C6—H6	119.0
N1^ii^—Sr1—C1^ii^	24.69 (4)	C7—C6—C5	122.03 (16)
N1^ii^—Sr1—C5^i^	68.71 (4)	C7—C6—H6	119.0
N2^i^—Sr1—Sr1^i^	81.44 (3)	O7—C7—C8	110.95 (16)
N2^i^—Sr1—O2^ii^	65.98 (4)	C6—C7—O7	121.31 (15)
N2^i^—Sr1—O6^i^	47.42 (4)	C6—C7—C8	127.71 (17)
N2^i^—Sr1—N1^ii^	82.54 (4)	C7—C8—H8A	109.5
N2^i^—Sr1—C1^ii^	67.66 (4)	C7—C8—H8B	109.5
N2^i^—Sr1—C5^i^	25.07 (4)	C7—C8—H8C	109.5
C1^ii^—Sr1—Sr1^i^	85.22 (3)	H8A—C8—H8B	109.5
C5^i^—Sr1—Sr1^i^	57.18 (3)	H8A—C8—H8C	109.5
C5^i^—Sr1—C1^ii^	63.40 (4)	H8B—C8—H8C	109.5
O4—S1—O3	106.58 (8)		
			
Sr1—O2—C1—Sr1^ii^	−151.3 (3)	O3—S1—N1—C1	−35.06 (15)
Sr1—O2—C1—N1	−175.1 (2)	O4—S1—O3—C3	−82.71 (14)
Sr1^ii^—O2—C1—N1	−23.76 (15)	O4—S1—O5—Sr1^iv^	170.15 (11)
Sr1—O2—C1—C2	−1.4 (4)	O4—S1—N1—Sr1^ii^	−126.76 (12)
Sr1^ii^—O2—C1—C2	149.95 (14)	O4—S1—N1—C1	81.76 (15)
Sr1—O6—C5—Sr1^i^	−162.9 (3)	O5—S1—O3—C3	155.40 (13)
Sr1^i^—O6—C5—N2	−20.16 (16)	O5—S1—N1—Sr1^ii^	5.17 (15)
Sr1—O6—C5—N2	177.0 (2)	O5—S1—N1—C1	−146.30 (13)
Sr1—O6—C5—C6	−7.8 (4)	O6—C5—C6—C7	−165.34 (17)
Sr1^i^—O6—C5—C6	155.04 (14)	O7—S2—O9—Sr1^iii^	−82.06 (12)
Sr1^ii^—N1—C1—O2	25.31 (16)	O7—S2—N2—Sr1^i^	123.73 (13)
Sr1^ii^—N1—C1—C2	−148.50 (13)	O7—S2—N2—C5	−35.08 (15)
Sr1^i^—N2—C5—O6	20.65 (16)	O8—S2—O7—C7	−79.80 (14)
Sr1^i^—N2—C5—C6	−154.58 (13)	O8—S2—O9—Sr1^iii^	161.68 (10)
Sr1^ii^—C1—C2—C3	−67.2 (3)	O8—S2—N2—Sr1^i^	−119.74 (13)
Sr1^i^—C5—C6—C7	−73.7 (4)	O8—S2—N2—C5	81.44 (15)
S1—O3—C3—C2	−19.8 (2)	O9—S2—O7—C7	157.64 (12)
S1—O3—C3—C4	164.84 (13)	O9—S2—N2—Sr1^i^	12.10 (16)
S1—N1—C1—Sr1^ii^	161.78 (14)	O9—S2—N2—C5	−146.71 (13)
S1—N1—C1—O2	−172.91 (13)	N1—S1—O3—C3	38.52 (15)
S1—N1—C1—C2	13.3 (2)	N1—S1—O5—Sr1^iv^	39.33 (14)
S2—O7—C7—C6	−24.5 (2)	N1—C1—C2—C3	11.3 (3)
S2—O7—C7—C8	157.25 (14)	N2—S2—O7—C7	40.92 (14)
S2—N2—C5—Sr1^i^	167.42 (14)	N2—S2—O9—Sr1^iii^	31.28 (14)
S2—N2—C5—O6	−171.93 (13)	N2—C5—C6—C7	9.7 (3)
S2—N2—C5—C6	12.8 (2)	C1—C2—C3—O3	−7.2 (3)
O2—C1—C2—C3	−162.30 (17)	C1—C2—C3—C4	167.20 (19)
O3—S1—O5—Sr1^iv^	−74.19 (13)	C5—C6—C7—O7	−3.0 (3)
O3—S1—N1—Sr1^ii^	116.42 (12)	C5—C6—C7—C8	175.01 (18)

Symmetry codes: (i) −*x*+2, −*y*+1, −*z*+1; (ii) −*x*+1, −*y*+1, −*z*+1; (iii) *x*+1, *y*, *z*; (iv) *x*−1, *y*, *z*.

### Poly[aquabis(µ3-6-methyl-2,2-dioxo-1,2λ6,3-oxathiazin-4-olato)strontium(II)] (Sr) Hydrogen-bond geometry (Å, º)

**Table:**

*D*—H···*A*	*D*—H	H···*A*	*D*···*A*	*D*—H···*A*
O1—H1*A*···O4^v^	0.79 (3)	2.18 (3)	2.899 (2)	151 (3)
O1—H1*B*···O8^vi^	0.83 (3)	2.22 (3)	2.9850 (19)	152 (3)
C4—H4*B*···O5^v^	0.98	2.37	3.273 (3)	153
C6—H6···O8^vi^	0.95	2.47	3.318 (2)	148

Symmetry codes: (v) *x*+1/2, −*y*+1/2, *z*−1/2; (vi) −*x*+2, −*y*+1, −*z*.

### Aquabis(µ3-6-methyl-2,2-dioxo-1,2λ6,3-oxathiazin-4-olato)barium(II) (Ba) Crystal data

**Table:**

[Ba(C_4_H_4_NO_4_S)_2_(H_2_O)]	*F*(000) = 928
*M**_r_* = 479.64	*D*_x_ = 2.104 Mg m^−^^3^
Monoclinic, *P*2_1_/*n*	Mo *K*α radiation, λ = 0.71073 Å
*a* = 8.2086 (5) Å	Cell parameters from 9794 reflections
*b* = 18.8899 (11) Å	θ = 3.0–36.3°
*c* = 9.8999 (6) Å	µ = 2.94 mm^−^^1^
β = 99.5197 (10)°	*T* = 173 K
*V* = 1513.93 (16) Å^3^	Block, colourless
*Z* = 4	0.52 × 0.21 × 0.20 mm

### Aquabis(µ3-6-methyl-2,2-dioxo-1,2λ6,3-oxathiazin-4-olato)barium(II) (Ba) Data collection

**Table:**

Bruker PHOTON-100 CMOS diffractometer	5218 reflections with *I* > 2σ(*I*)
Radiation source: sealedtube	*R*_int_ = 0.048
φ and ω scans	θ_max_ = 34.3°, θ_min_ = 3.0°
Absorption correction: numerical (SADABS; Krause *et al.*, 2015)	*h* = −13→13
*T*_min_ = 0.309, *T*_max_ = 0.635	*k* = −29→29
41654 measured reflections	*l* = −15→15
6324 independent reflections	

### Aquabis(µ3-6-methyl-2,2-dioxo-1,2λ6,3-oxathiazin-4-olato)barium(II) (Ba) Refinement

**Table:**

Refinement on *F*^2^	Primary atom site location: isomorphous structure methods
Least-squares matrix: full	Hydrogen site location: mixed
*R*[*F*^2^ > 2σ(*F*^2^)] = 0.032	H atoms treated by a mixture of independent and constrained refinement
*wR*(*F*^2^) = 0.059	*w* = 1/[σ^2^(*F*_o_^2^) + (0.0158*P*)^2^ + 2.2204*P*] where *P* = (*F*_o_^2^ + 2*F*_c_^2^)/3
*S* = 1.09	(Δ/σ)_max_ = 0.001
6324 reflections	Δρ_max_ = 1.02 e Å^−^^3^
209 parameters	Δρ_min_ = −0.61 e Å^−^^3^
0 restraints	

### Aquabis(µ3-6-methyl-2,2-dioxo-1,2λ6,3-oxathiazin-4-olato)barium(II) (Ba) Special details

**Table:**

Geometry. All esds (except the esd in the dihedral angle between two l.s. planes) are estimated using the full covariance matrix. The cell esds are taken into account individually in the estimation of esds in distances, angles and torsion angles; correlations between esds in cell parameters are only used when they are defined by crystal symmetry. An approximate (isotropic) treatment of cell esds is used for estimating esds involving l.s. planes.

### Aquabis(µ3-6-methyl-2,2-dioxo-1,2λ6,3-oxathiazin-4-olato)barium(II) (Ba) Fractional atomic coordinates and isotropic or equivalent isotropic displacement parameters (Å^2^)

**Table:**

	*x*	*y*	*z*	*U*_iso_*/*U*_eq_	
Ba1	0.73689 (2)	0.46819 (2)	0.43614 (2)	0.01376 (3)	
S1	0.07538 (7)	0.32148 (3)	0.54490 (6)	0.01768 (10)	
S2	1.36455 (6)	0.52303 (3)	0.15371 (6)	0.01847 (11)	
O1	0.6927 (3)	0.37493 (12)	0.2222 (2)	0.0313 (4)	
H1A	0.692 (5)	0.336 (2)	0.213 (4)	0.047 (12)*	
H1B	0.664 (5)	0.389 (2)	0.153 (4)	0.041 (11)*	
O2	0.4531 (2)	0.40981 (10)	0.48151 (19)	0.0255 (4)	
O3	0.0469 (2)	0.28363 (10)	0.39659 (19)	0.0259 (4)	
O4	0.1222 (3)	0.26718 (10)	0.6433 (2)	0.0314 (4)	
O5	−0.0809 (2)	0.35404 (9)	0.5498 (2)	0.0260 (4)	
O6	1.0152 (2)	0.48791 (10)	0.33283 (18)	0.0227 (4)	
O7	1.3411 (2)	0.44828 (10)	0.07381 (19)	0.0270 (4)	
O8	1.3107 (2)	0.57651 (11)	0.05494 (18)	0.0270 (4)	
O9	1.5363 (2)	0.52380 (11)	0.21146 (18)	0.0257 (4)	
N1	0.2129 (2)	0.37885 (10)	0.5425 (2)	0.0190 (4)	
N2	1.2552 (2)	0.51888 (11)	0.27018 (19)	0.0190 (4)	
C1	0.3361 (3)	0.36655 (12)	0.4691 (2)	0.0185 (4)	
C2	0.3227 (3)	0.30894 (13)	0.3700 (3)	0.0240 (5)	
H2	0.416716	0.296609	0.330515	0.029*	
C3	0.1826 (3)	0.27297 (13)	0.3330 (3)	0.0233 (5)	
C4	0.1416 (4)	0.22233 (17)	0.2179 (3)	0.0381 (7)	
H4A	0.104320	0.177552	0.252407	0.057*	
H4B	0.239932	0.213846	0.175937	0.057*	
H4C	0.053596	0.242128	0.149386	0.057*	
C5	1.1101 (3)	0.48350 (12)	0.2459 (2)	0.0169 (4)	
C6	1.0711 (3)	0.43709 (13)	0.1272 (2)	0.0213 (4)	
H6	0.962455	0.418511	0.104690	0.026*	
C7	1.1828 (3)	0.41992 (14)	0.0493 (2)	0.0237 (5)	
C8	1.1637 (5)	0.36907 (18)	−0.0670 (3)	0.0422 (8)	
H8A	1.184568	0.393477	−0.149831	0.063*	
H8B	1.051152	0.350013	−0.082391	0.063*	
H8C	1.242929	0.330240	−0.045421	0.063*	

### Aquabis(µ3-6-methyl-2,2-dioxo-1,2λ6,3-oxathiazin-4-olato)barium(II) (Ba) Atomic displacement parameters (Å^2^)

**Table:**

	*U*^11^	*U*^22^	*U*^33^	*U*^12^	*U*^13^	*U*^23^
Ba1	0.01155 (5)	0.01638 (6)	0.01386 (6)	−0.00196 (5)	0.00359 (4)	−0.00155 (5)
S1	0.0171 (2)	0.0136 (2)	0.0238 (3)	0.00023 (19)	0.0076 (2)	0.0012 (2)
S2	0.0133 (2)	0.0298 (3)	0.0126 (2)	−0.0002 (2)	0.00297 (18)	0.0006 (2)
O1	0.0517 (13)	0.0203 (10)	0.0211 (10)	−0.0050 (9)	0.0039 (9)	−0.0042 (8)
O2	0.0182 (8)	0.0281 (9)	0.0304 (10)	−0.0065 (7)	0.0049 (7)	0.0006 (8)
O3	0.0212 (8)	0.0291 (9)	0.0286 (9)	−0.0067 (7)	0.0076 (7)	−0.0093 (8)
O4	0.0402 (11)	0.0195 (9)	0.0348 (11)	0.0034 (8)	0.0072 (9)	0.0098 (8)
O5	0.0188 (8)	0.0224 (8)	0.0397 (11)	0.0023 (7)	0.0135 (7)	0.0028 (8)
O6	0.0171 (7)	0.0319 (9)	0.0211 (8)	−0.0024 (7)	0.0093 (6)	−0.0025 (7)
O7	0.0230 (8)	0.0367 (10)	0.0232 (9)	0.0001 (7)	0.0098 (7)	−0.0092 (8)
O8	0.0237 (8)	0.0377 (11)	0.0195 (9)	0.0005 (8)	0.0026 (7)	0.0089 (8)
O9	0.0139 (7)	0.0438 (11)	0.0193 (8)	−0.0010 (7)	0.0023 (6)	0.0032 (8)
N1	0.0163 (8)	0.0172 (9)	0.0244 (10)	−0.0023 (7)	0.0065 (7)	−0.0016 (7)
N2	0.0154 (8)	0.0290 (11)	0.0131 (8)	−0.0037 (7)	0.0045 (7)	−0.0021 (7)
C1	0.0146 (9)	0.0190 (10)	0.0217 (11)	0.0016 (8)	0.0021 (8)	0.0032 (8)
C2	0.0227 (11)	0.0232 (12)	0.0282 (13)	0.0035 (9)	0.0109 (10)	−0.0014 (10)
C3	0.0263 (12)	0.0225 (11)	0.0220 (11)	0.0014 (9)	0.0072 (9)	−0.0030 (9)
C4	0.0475 (18)	0.0343 (15)	0.0342 (16)	−0.0044 (13)	0.0123 (14)	−0.0140 (13)
C5	0.0154 (9)	0.0202 (10)	0.0153 (10)	0.0007 (8)	0.0028 (8)	0.0020 (8)
C6	0.0197 (10)	0.0264 (12)	0.0172 (11)	−0.0047 (9)	0.0014 (8)	−0.0023 (9)
C7	0.0283 (12)	0.0256 (12)	0.0171 (11)	−0.0021 (10)	0.0036 (9)	−0.0017 (9)
C8	0.058 (2)	0.0421 (18)	0.0293 (15)	−0.0127 (15)	0.0151 (14)	−0.0172 (13)

### Aquabis(µ3-6-methyl-2,2-dioxo-1,2λ6,3-oxathiazin-4-olato)barium(II) (Ba) Geometric parameters (Å, º)

**Table:**

Ba1—Ba1^i^	4.4491 (3)	O1—H1B	0.74 (4)
Ba1—O1	2.732 (2)	O2—C1	1.252 (3)
Ba1—O2^i^	2.9714 (19)	O3—C3	1.382 (3)
Ba1—O2	2.6812 (17)	O6—C5	1.256 (3)
Ba1—O5^ii^	2.7560 (18)	O7—C7	1.389 (3)
Ba1—O6	2.6788 (16)	N1—C1	1.359 (3)
Ba1—O6^iii^	2.9192 (18)	N2—C5	1.352 (3)
Ba1—O9^iv^	2.7478 (18)	C1—C2	1.457 (3)
Ba1—N1^i^	2.921 (2)	C2—H2	0.9500
Ba1—N2^iii^	2.9077 (19)	C2—C3	1.334 (4)
Ba1—C1^i^	3.342 (2)	C3—C4	1.483 (4)
Ba1—C5^iii^	3.318 (2)	C4—H4A	0.9800
S1—O3	1.6149 (19)	C4—H4B	0.9800
S1—O4	1.4232 (19)	C4—H4C	0.9800
S1—O5	1.4309 (17)	C5—C6	1.459 (3)
S1—N1	1.568 (2)	C6—H6	0.9500
S2—O7	1.6143 (19)	C6—C7	1.332 (3)
S2—O8	1.4245 (19)	C7—C8	1.488 (4)
S2—O9	1.4313 (17)	C8—H8A	0.9800
S2—N2	1.5758 (19)	C8—H8B	0.9800
O1—H1A	0.75 (4)	C8—H8C	0.9800
			
O1—Ba1—Ba1^i^	112.59 (5)	O4—S1—O5	115.39 (12)
O1—Ba1—O2^i^	134.76 (6)	O4—S1—N1	113.25 (12)
O1—Ba1—O5^ii^	78.79 (7)	O5—S1—O3	103.03 (11)
O1—Ba1—O6^iii^	140.94 (6)	O5—S1—N1	110.81 (11)
O1—Ba1—O9^iv^	68.53 (7)	N1—S1—O3	106.77 (10)
O1—Ba1—N1^i^	133.85 (6)	O8—S2—O7	106.60 (11)
O1—Ba1—N2^iii^	143.83 (6)	O8—S2—O9	116.14 (11)
O1—Ba1—C1^i^	144.37 (7)	O8—S2—N2	112.82 (11)
O1—Ba1—C5^iii^	153.32 (7)	O9—S2—O7	103.55 (11)
O2^i^—Ba1—Ba1^i^	35.84 (3)	O9—S2—N2	110.54 (10)
O2—Ba1—Ba1^i^	40.45 (4)	N2—S2—O7	106.20 (10)
O2—Ba1—O1	81.78 (6)	Ba1—O1—H1A	137 (3)
O2—Ba1—O2^i^	76.29 (6)	Ba1—O1—H1B	118 (3)
O2—Ba1—O5^ii^	92.16 (5)	H1A—O1—H1B	105 (4)
O2—Ba1—O6^iii^	119.87 (5)	Ba1—O2—Ba1^i^	103.71 (6)
O2—Ba1—O9^iv^	82.65 (5)	C1—O2—Ba1	157.81 (16)
O2—Ba1—N1^i^	120.71 (5)	C1—O2—Ba1^i^	95.98 (14)
O2—Ba1—N2^iii^	75.13 (5)	C3—O3—S1	118.19 (16)
O2^i^—Ba1—C1^i^	21.87 (5)	S1—O5—Ba1^iv^	138.72 (10)
O2—Ba1—C1^i^	97.82 (6)	Ba1—O6—Ba1^iii^	105.57 (5)
O2—Ba1—C5^iii^	99.04 (5)	C5—O6—Ba1^iii^	97.13 (14)
O2^i^—Ba1—C5^iii^	70.11 (5)	C5—O6—Ba1	156.59 (16)
O5^ii^—Ba1—Ba1^i^	122.42 (4)	C7—O7—S2	117.07 (15)
O5^ii^—Ba1—O2^i^	140.25 (5)	S2—O9—Ba1^ii^	137.59 (10)
O5^ii^—Ba1—O6^iii^	68.99 (5)	S1—N1—Ba1^i^	140.88 (10)
O5^ii^—Ba1—N1^i^	133.14 (6)	C1—N1—Ba1^i^	95.70 (13)
O5^ii^—Ba1—N2^iii^	74.73 (6)	C1—N1—S1	119.41 (17)
O5^ii^—Ba1—C1^i^	136.60 (6)	S2—N2—Ba1^iii^	143.73 (10)
O5^ii^—Ba1—C5^iii^	74.53 (6)	C5—N2—Ba1^iii^	95.30 (13)
O6—Ba1—Ba1^i^	155.73 (4)	C5—N2—S2	119.09 (16)
O6^iii^—Ba1—Ba1^i^	103.27 (3)	O2—C1—Ba1^i^	62.15 (13)
O6—Ba1—O1	78.82 (6)	O2—C1—N1	117.5 (2)
O6^iii^—Ba1—O2^i^	83.93 (5)	O2—C1—C2	121.7 (2)
O6—Ba1—O2	160.13 (6)	N1—C1—Ba1^i^	60.43 (12)
O6—Ba1—O2^i^	121.09 (5)	N1—C1—C2	120.5 (2)
O6—Ba1—O5^ii^	79.94 (5)	C2—C1—Ba1^i^	152.82 (16)
O6—Ba1—O6^iii^	74.43 (5)	C1—C2—H2	119.0
O6—Ba1—O9^iv^	94.15 (5)	C3—C2—C1	122.1 (2)
O6^iii^—Ba1—N1^i^	65.83 (5)	C3—C2—H2	119.0
O6—Ba1—N1^i^	76.77 (5)	O3—C3—C4	110.3 (2)
O6—Ba1—N2^iii^	119.39 (5)	C2—C3—O3	121.6 (2)
O6^iii^—Ba1—C1^i^	69.44 (5)	C2—C3—C4	128.0 (2)
O6—Ba1—C1^i^	100.43 (5)	C3—C4—H4A	109.5
O6—Ba1—C5^iii^	96.38 (5)	C3—C4—H4B	109.5
O6^iii^—Ba1—C5^iii^	22.06 (5)	C3—C4—H4C	109.5
O9^iv^—Ba1—Ba1^i^	72.06 (4)	H4A—C4—H4B	109.5
O9^iv^—Ba1—O2^i^	69.77 (5)	H4A—C4—H4C	109.5
O9^iv^—Ba1—O5^ii^	147.31 (6)	H4B—C4—H4C	109.5
O9^iv^—Ba1—O6^iii^	140.57 (6)	O6—C5—Ba1^iii^	60.81 (12)
O9^iv^—Ba1—N1^i^	74.86 (6)	O6—C5—N2	118.3 (2)
O9^iv^—Ba1—N2^iii^	133.52 (6)	O6—C5—C6	120.9 (2)
O9^iv^—Ba1—C1^i^	76.05 (6)	N2—C5—Ba1^iii^	60.77 (11)
O9^iv^—Ba1—C5^iii^	138.14 (6)	N2—C5—C6	120.65 (19)
N1^i^—Ba1—Ba1^i^	80.31 (4)	C6—C5—Ba1^iii^	158.10 (16)
N1^i^—Ba1—O2^i^	44.54 (5)	C5—C6—H6	118.9
N1^i^—Ba1—C1^i^	23.87 (5)	C7—C6—C5	122.1 (2)
N1^i^—Ba1—C5^iii^	68.44 (6)	C7—C6—H6	118.9
N2^iii^—Ba1—Ba1^i^	64.48 (4)	O7—C7—C8	111.1 (2)
N2^iii^—Ba1—O2^i^	65.57 (5)	C6—C7—O7	121.2 (2)
N2^iii^—Ba1—O6^iii^	45.20 (5)	C6—C7—C8	127.6 (3)
N2^iii^—Ba1—N1^i^	82.24 (6)	C7—C8—H8A	109.5
N2^iii^—Ba1—C1^i^	67.43 (6)	C7—C8—H8B	109.5
N2^iii^—Ba1—C5^iii^	23.94 (5)	C7—C8—H8C	109.5
C1^i^—Ba1—Ba1^i^	57.47 (4)	H8A—C8—H8B	109.5
C5^iii^—Ba1—Ba1^i^	82.39 (4)	H8A—C8—H8C	109.5
C5^iii^—Ba1—C1^i^	62.23 (6)	H8B—C8—H8C	109.5
O4—S1—O3	106.60 (11)		
			
Ba1—O2—C1—Ba1^i^	−152.6 (4)	O3—S1—N1—C1	−33.6 (2)
Ba1—O2—C1—N1	−177.7 (3)	O4—S1—O3—C3	−84.9 (2)
Ba1^i^—O2—C1—N1	−25.2 (2)	O4—S1—O5—Ba1^iv^	170.65 (15)
Ba1—O2—C1—C2	−3.7 (6)	O4—S1—N1—Ba1^i^	−125.67 (17)
Ba1^i^—O2—C1—C2	148.9 (2)	O4—S1—N1—C1	83.4 (2)
Ba1—O6—C5—Ba1^iii^	−165.9 (4)	O5—S1—O3—C3	153.20 (18)
Ba1^iii^—O6—C5—N2	−20.6 (2)	O5—S1—N1—Ba1^i^	5.8 (2)
Ba1—O6—C5—N2	173.5 (3)	O5—S1—N1—C1	−145.06 (19)
Ba1—O6—C5—C6	−11.1 (5)	O6—C5—C6—C7	−165.8 (2)
Ba1^iii^—O6—C5—C6	154.73 (19)	O7—S2—O9—Ba1^ii^	−80.89 (17)
Ba1^i^—N1—C1—O2	25.6 (2)	O7—S2—N2—Ba1^iii^	124.24 (18)
Ba1^i^—N1—C1—C2	−148.52 (19)	O7—S2—N2—C5	−35.3 (2)
Ba1^iii^—N2—C5—O6	20.6 (2)	O8—S2—O7—C7	−79.77 (19)
Ba1^iii^—N2—C5—C6	−154.73 (19)	O8—S2—O9—Ba1^ii^	162.65 (14)
Ba1^i^—C1—C2—C3	−74.1 (4)	O8—S2—N2—Ba1^iii^	−119.34 (18)
Ba1^iii^—C5—C6—C7	−77.8 (5)	O8—S2—N2—C5	81.1 (2)
S1—O3—C3—C2	−18.7 (3)	O9—S2—O7—C7	157.23 (18)
S1—O3—C3—C4	165.55 (19)	O9—S2—N2—Ba1^iii^	12.6 (2)
S1—N1—C1—Ba1^i^	162.0 (2)	O9—S2—N2—C5	−146.95 (19)
S1—N1—C1—O2	−172.35 (18)	N1—S1—O3—C3	36.4 (2)
S1—N1—C1—C2	13.5 (3)	N1—S1—O5—Ba1^iv^	40.3 (2)
S2—O7—C7—C6	−24.1 (3)	N1—C1—C2—C3	9.8 (4)
S2—O7—C7—C8	157.7 (2)	N2—S2—O7—C7	40.8 (2)
S2—N2—C5—Ba1^iii^	168.0 (2)	N2—S2—O9—Ba1^ii^	32.5 (2)
S2—N2—C5—O6	−171.40 (18)	N2—C5—C6—C7	9.4 (4)
S2—N2—C5—C6	13.3 (3)	C1—C2—C3—O3	−6.5 (4)
O2—C1—C2—C3	−164.1 (2)	C1—C2—C3—C4	168.5 (3)
O3—S1—O5—Ba1^iv^	−73.61 (18)	C5—C6—C7—O7	−3.1 (4)
O3—S1—N1—Ba1^i^	117.32 (17)	C5—C6—C7—C8	174.7 (3)

Symmetry codes: (i) −*x*+1, −*y*+1, −*z*+1; (ii) *x*+1, *y*, *z*; (iii) −*x*+2, −*y*+1, −*z*+1; (iv) *x*−1, *y*, *z*.

### Aquabis(µ3-6-methyl-2,2-dioxo-1,2λ6,3-oxathiazin-4-olato)barium(II) (Ba) Hydrogen-bond geometry (Å, º)

**Table:**

*D*—H···*A*	*D*—H	H···*A*	*D*···*A*	*D*—H···*A*
O1—H1*A*···O4^v^	0.74 (4)	2.12 (4)	2.829 (3)	162 (4)
O1—H1*B*···O8^vi^	0.74 (4)	2.20 (4)	2.889 (3)	156 (4)
C4—H4*B*···O5^v^	0.98	2.44	3.361 (4)	155
C6—H6···O8^vi^	0.95	2.52	3.357 (3)	147

Symmetry codes: (v) *x*+1/2, −*y*+1/2, *z*−1/2; (vi) −*x*+2, −*y*+1, −*z*.
